# miR‐21 modification enhances the performance of adipose tissue‐derived mesenchymal stem cells for counteracting urethral stricture formation

**DOI:** 10.1111/jcmm.13834

**Published:** 2018-09-04

**Authors:** Zongcheng Feng, Hongrun Chen, Taozhu Fu, Lianfeng Zhang, Yushan Liu

**Affiliations:** ^1^ Department of Urology No. 731 Hospital of China Aerospace Science and Industry Corporation Beijing China

**Keywords:** ADSCs, miR‐21 modification, urethral stricture, wound healing

## Abstract

The treatment of complicated long segment strictures remains to a challenge, and the substitution urethroplasty treatment is often accompanied by subsequent tissue fibrosis and secondary stricture formation. In situ injection of human adipose tissue‐derived stem cells (hADSC) could potential be applied for prevention of urethral fibrosis, but the cells transplantation alone may be insufficient because of the complicated histopathological micro‐environmental changes in the injury site. This study investigated whether miR‐21 modification can improve the therapeutic efficacy of ADSCs against urethral fibrosis to limit urethral stricture recurrence. MiR‐21‐modified ADSCs (miR‐21) were constructed via lentivirus‐mediated transfer of pre‐miR‐21 and GFP reporter gene. In vitro results suggested that miR‐21 modification can increase the angiogenesis genes expression of ADSCs and enhance its anti‐oxidative effects against reactive oxygen species (ROS) damage. In vivo results showed that miR‐21 modification contributes to increased urodynamic parameters and better formation of the epithelium and the muscle layer as compared to ADSCs transplantation alone groups. The results demonstrated that miR‐21 modification in ADSCs could improve urethral wound healing microenvironment, enhance stem cell survival through ROS scavenging and promote the neovascularization via regulating angiogenic genes expression, which eventually increase the ADSCs' therapeutic potential for urethral wound healing.

## 1 INTRODUCTION

Despite considerable progress in the modern medicine and surgery, the treatment of complicated long segment strictures is still very difficult and remains to be one of the challenges.^1^, ^2^ Normally, the performance of current surgical repair techniques such as urethroplasty or direct end‐to‐end anastomosis is often accompanied by subsequent tissue fibrosis, poor vascularization and chronic inflammation, which might eventually lead to secondary stricture formation.^3^, ^4^ The impaired wound healing after urethroplasty is proved to be a complex biological process, during which the inflammation, tissue formation including angiogenesis and granulation, and tissue remodelling play vital roles.^5^ Thus multiple impairments of cellular responses contributed to non‐healing wounds during the wound healing process after urethroplasty, which make it a more complicated issue for success management of urethral stricture disease. Although the administration of anti‐fibrotic drugs like halofuginone has been proved to limit recurrence of urethral stricture, none of these medicines was observed with sufficient therapeutic benefit.^6^

Recently various strategies, including growth factors administration, gene therapy, and stem cell transplantation, have been used to improve the treatment of urethral stricture.^4^, ^7^, ^8^, ^9^ Among these, the transplantation of mesenchymal stem cells (MSC)—the multipotent stromal progenitor cells—has been proved to enhance the tissue repair process and resulted in better performance.^10^, ^11^ Large number of papers indicated that both multi‐potential ability to differentiate into specialized cells for injury replacement and its paracrine effects to regulate the wound healing process, contribute to high therapeutic potential of MSCs during tissue repair or regeneration process.^7^, ^8^, ^9^ However, because of the unfavourable microenvironment in the repair site, such as inflammation and over‐production of ROS induced oxidative stress, the poor viability of MSC at the transplanted site might significantly decreased its therapeutic potential performance.^12^, ^13^ It is important to improve the survival of transplanted MSCs and enhance the biological functions in vivo. Recently, many studies have tried to increase the repair performance of transplanted MSCs via various intervention methods, including gene modification and biomaterials‐based tissue engineering approaches. In particular, increasing evidences proved that microRNAs (miRNAs)‐based gene modification of MSCs is proved to have great potential for regenerative medicine.^14^, ^15^, ^16^

The evolutionary conserved miRNAs with length of approximately 20‐24 nucleotide played important and various roles in the control of genes expression by negatively regulating the translation of target genes expression.^17^, ^18^ As so far, miRNAs are proved to be implicated in many processes of biology metabolism, including the cellular proliferation and apoptosis, neuronal patterning and tumorigenesis, etc. What's more important, the miRNAs also evolved or directly regulated many complicated cell fate decisions and diseases via interfering a large number of regulated genes. Among these, the miR‐21 was reported with multi‐faceted regulation effects, especially in stem cell biology. Emerging studies indicated that miR‐21 might contribute to the self‐renewal, lineage differentiation and paracrine effects of MSCs.^19^, ^20^, ^21^, ^22^ Recently studies have also shown that microRNA‐21 may play pivotal roles in the regulation of a variety of skin fibrosis, including keloid, and hypertrophic scar.^20^, ^23^ These studies indicated the potential application of miRNA‐21 modification for enhanced MSC‐based (therapeutic potential) regenerative purpose in the counteracting of urethral stricture formation.

To address this issue, we hypothesize that the miR‐21 modification of human adipose‐derived mesenchymal stem cells (ADSCs) might be able to enhance its therapeutic potential for the healing and reconstitution of the urethra postoperatively to limit urethral stricture recurrence. The lentiviral‐based transfection was used to enhance the expression of miR‐21, and the therapeutic potential of MSCs was also evaluated in vitro and in vivo as well.

## 2 MATERIALS AND METHODS

### 2.1 Cultivation and characterization of human ADSCs

The human ADSCs were bought from Cyagen Bioscience Inc Company (Suzhou, China) and cultured in a‐MEM (GIBCO, Invitrogen, California, USA) supplemented with 10% foetal bovine serum (FBS), 1 mmol/L L‐glutamine, and 1% penicillin/streptomycin (Invitrogen, California, USA). To characterize the immunophenotype of ADSCs, surface markers Cluster of Differentiation 105 (CD105), Cluster of Differentiation 90 (CD90), and Cluster of Differentiation 45 (CD45) were analyzed via flow cytometry.

### 2.2 Lentiviral based transfection of ADSCs with miR‐21

The miR‐21 lentiviral vector was generated by sub‐cloning the precursor miR‐21 fragment into the Pac1/Nhe1 cloning sites of the plasmid FUGW, a self‐inactivating, replication incompetent lentiviral vector that carries the human ubiquitin‐C promoter driving an enhanced green fluorescent protein (GFP) reporter gene. Human embryonic kidney 293T cells were transfected using SuperFect Transfection Reagent (Qiagen, Hilden, Germany) with the expression system vectors. After that, the virions were isolated by ultracentrifugation of filtered supernatant and applied for transfection (Lenti‐miR‐21 group). The vector without the miR‐21 insert was used as negative group (Lenti‐GFP group).

Adipose‐derived mesenchymal stem cells were transfected with lentiviral at third passage (P3). The efficiency of lentiviral gene transfer in ADSCs was optimize, and the highest transfection efficiency was applied for the following experiments. After 72 hours, the expression of GFP marker gene was observed using an Olympas inverted microscope system (Olympus IX71, Olympus, Tokyo, Japan), and flow cytometry to indicate the transfection efficiency. The cell viability at firth, second and third day after transfection was measured by a Cell Counting Kit‐8 kit (CCK‐8) according to manufacturer's instructions.

### 2.3 qRT‐PCR analysis

Seventy‐two hours after transfection, total RNA of the lentiviral vector transfected ADSCs was extracted using a total RNA pure Kit (Omega) according to manufacturer's instruction. The first‐strand complementary DNA (cDNA) was obtained by reverse transcription via using a PrimeScript RT reagent Kit with gDNA Eraser (TaKaRa, Dalian, China). The expression of angiogenesis‐related genes was detected by quantitative RT‐PCR, and the mRNA levels were normalized to the endogenous reference gene GADPH. The gene‐specific primers were designed by PubMed website and listed in Table S1. The PCR was performed with a hot start at 95.0°C for 45 seconds, followed by 40 cycles at 95.0°C for 5 seconds and 60.0°C for 30 seconds. The experiments were repeated for three times.

### 2.4 Western blotting

Seventy‐two hours fter transfection, the cells were lysed in Laemmli Sample Buffer (Bio‐Rad, Hercules, CA, USA) and the total proteins concentrations were quantitative measured using the BCA Protein Assay Kit (Beyotime, Beijing, China). Equal amounts (80 μg) of total proteins were loaded on a 12% SDS‐polyacrylamide gel and separated by electrophoresis. After being transferred to a PVDF membrane, the sample was blocked with 5% defatted milk and followed by primary antibody incubation overnight at 4°C. The primary antibodies hypoxia‐inducible factor 1‐alpha (HIF‐1α), vascular endothelial growth factor (VEGF), basic fibroblast growth factor (bFGF), hepatocyte growth factor‐1 (HGF‐1), stem cell factor (SCF), stromal cell‐derived factor‐1(SDF‐1) and d‐glyceraldehyde‐3‐phosphate dehydrogenase (GADPH) from Cell Signalling Technology (Beverly, MA, USA) were used. The proteins were then incubated with horseradish peroxidase‐conjugated secondary antibodies, and the signals of protein bands were detected by enhanced chemiluminescence reagent (Applygen, Beijing, China). Band intensity was normalized with GADPH as the endogenous control.

### 2.5 Anti‐oxidative protection role of miR21 on ADSCs

In order to further evaluate the anti‐oxidative protection role of miR21 on ADSCs, the hydrogen peroxide (H_2_O_2_)‐treatment was used as the exogenous ROS source to induce oxidative stress microenvironment. In shortly, when the cells were split at 70%‐80% confluence, the growth medium was replaced with different concentrations of H_2_O_2_ (0‐80 μmol/L) and cultured for further 24 hours. The cell viability was measured by a Cell Counting Kit‐8 kit (CCK‐8) according to manufacturer's instructions.

To measure the percentage of dead cell, the cells treated with 60 μmol/L H_2_O_2_ were collected and analysed by live/dead staining according to manufacturer's instruction. In order to analyse the apoptotic cells, the cell was fixed with 4% paraformaldehyde for 20 minutes and then terminal deoxyribonucleotidyl transferse (TdT)‐mediated biotin‐16‐dUTP nick‐end labelling (TUNEL) staining was performed according to the manufacturer's instructions (Beyotime). The results were observed using a fluorescent microscopy (Olympus), and fluorescence images were obtained. The percentage of TUNEL‐positive cells was calculated accordingly, and six samples for each group were included. For each sample, five random high resolution fields were applied and counted in a blinded manner.

Twenty‐four hours after the 60 μmol/L H_2_O_2_ treatment, total RNA of the lentiviral vector transfected ADSCs was extracted and the mRNA expression levels of B‐cell lymphoma‐2 (Bcl‐2) and Bax were detected via PCR amplification using Pfu PCR MasterMix (Tiangen, Beijing, China) and normalized to the endogenous reference gene β‐Actin. The gene‐specific primers were designed by PubMed website and listed in Table S1.

### 2.6 In vivo transplantation of ADSCs for US model

The Sprague‐Dawley rats were applied, and all animal experiments were conducted with the approval of the Ethics Committee of Animal Experiments of Beijing, China. The rats were anesthetized with pentobarbital sodium at a dose of 30 mg/kg and randomly divided into four groups. The rats received a methylene blue marked catheter and blood flow was blocked using elastic tourniquet. The ventral penile skin was exposed and 100 μL of saline (US group) or ADSCs suspension (1 × 10^6^) was injected into the urethral wall at four different sites semi‐circumferentially along 0.8‐1.0 cm of the exposed urethra with a 30G needle. 5‐10 minutes after the injections, the rats underwent four partial incisions of the penile urethra with a 23G needle the rats. All layers of the urethral wall were surgical cut to visualize the catheter. After removing the catheter, the penile skin was sutured up with absorbable sutures. The rats injected with cell suspension of MSCs, the MSCs transfected with only marker genes GFP lentiviral vector and the MSCs transfected with miR‐21 lentiviral vector were denoted as MSC, GFP and miR‐21 groups, respectively (n = 12).

### 2.7 In vivo functional and histological examination

Four weeks later, urodynamic parameters were measured. Briefly, the rats were firstly anaesthetized with chloral hydrate (350 mg/kg) and held under partial restraint in a restraining device. The bladder was catheterized through urethra by polyethylene catheter (0.7 mm outer diameter, 0.4 mm internal diameter). The catheter was to urodynamic testing machine and infusion pump via a T‐tube, and the conscious rats underwent cytometry for micturition volume (mL) and maximum flow pressure (cm‐H_2_O) parameters. To further measure the urethral diameter, the micro‐ultrasound was performed using a Vevo 2100 micro‐ultrasound imaging system (Visual‐ Sonics Inc., Toronto, ON, Canada) with a 40 MHz linear‐array transducer (MS‐550D). The urethral diameter was calculated accordingly at the injury site and six rats were included for each group.

The rats were then immediately euthanized to obtain the bladder, penile and urethral tissues for histological examination and Western blotting. As for histological examination, the tissues were dehydrated, cleared and embedded in paraffin as normal. Three‐ to five‐micrometer slices were obtained for Masson's trichrome and von Willebrand Factor (vWF) staining. Images were observed with Olympus IX71 inverted microscope.

### 2.8 In vivo RT‐PCR and Western blotting

To analyse the mRNA expression of angiogenic relative genes in transplanted human ADSCs, total RNA of tissue was extracted and the expression of angiogenesis related human genes was detected by quantitative RT‐PCR as described previously in 2.3. To measure the proteins extents, the tissues were lysed in Laemmli Sample Buffer (Bio‐Rad) and the total proteins obtained. The proteins extents of HIF‐1α, VEGF, endothelial nitric oxide synthase (eNOS), inducible nitric oxide synthase (iNOS), Collagen III, Elastin and β‐Actin (Cell Signaling Technology) were detected as described previously in 2.4, and the band intensity was normalized with β‐Actin as the endogenous control.

### 2.9 Statistical analysis

All data were expressed a mean ± SD (standard error). One‐way ANOVA followed by the Student‐Newman‐Keuls test for post hoc comparisons were used to evaluate the statistical significance with *P*‐values of 0.05 and 0.01. Statistical analysis was performed using SPSS Statistics (version 19.0, IBM Co., Chicago, IL).

## 3 RESULTS

### 3.1 miR‐21 increase the angiogenesis genes expression of ADSCs

**Figure 1:**
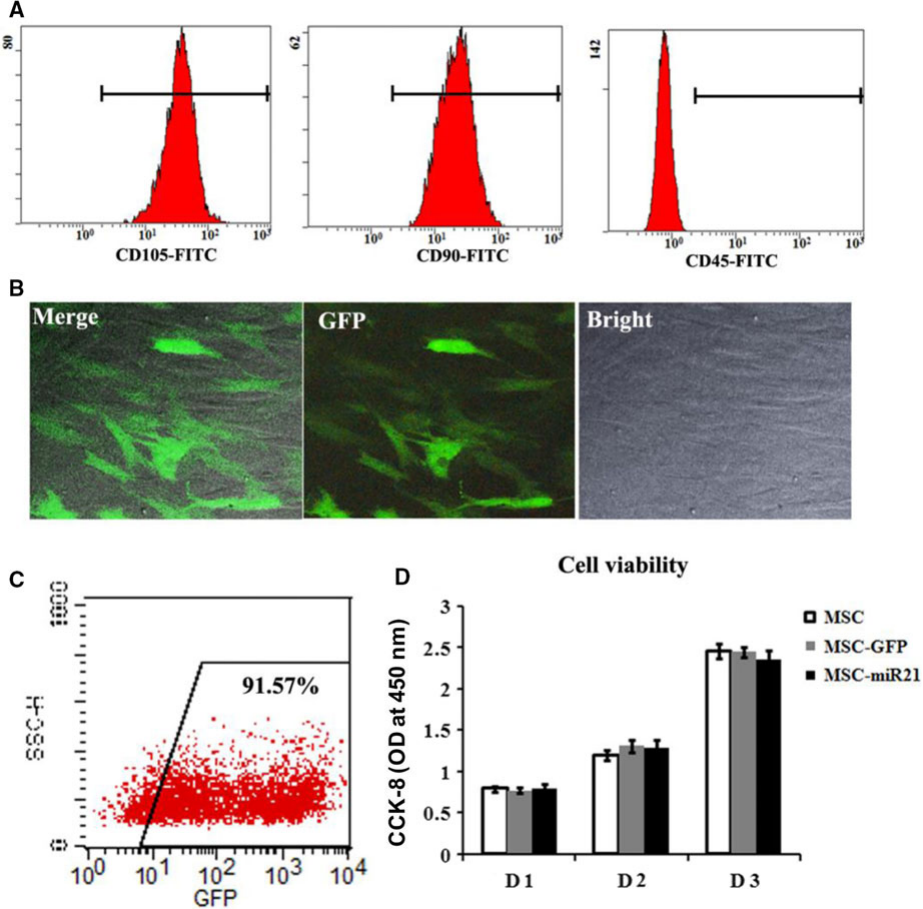
miR‐21 modification of ADSCs via lentiviral‐based transfection. A, Flow cytometric analysis of the surface markers in P3 ADSCs. B, GFP marker gene expression and cellular morphology of miR‐21‐modified ADSCs. C, Flow cytometric analysis of the GFP expression in miR‐21‐modified ADSCs. D, In vitro cell viability of ADSCs; *P < 0.05, **P < 0.01

The flow cytometry was performed to characterize the expression of the cellular surface proteins. As shown in Figure 1A, the cells after three passages mostly expressed MSC markers CD105 and CD90, but were negative for heamatopoietic‐specific marker CD45. After being transfected with lentiviral vector, most of the ADSCs (over 90%) were observed with GFP expression, which was further confirmed by flow cytometry (Figure 1B,C). CCK‐8 results suggested that modification of the ADSCs via lentiviral transfection has no significant impact on the cell viability of ADSCs.

**Figure 2:**
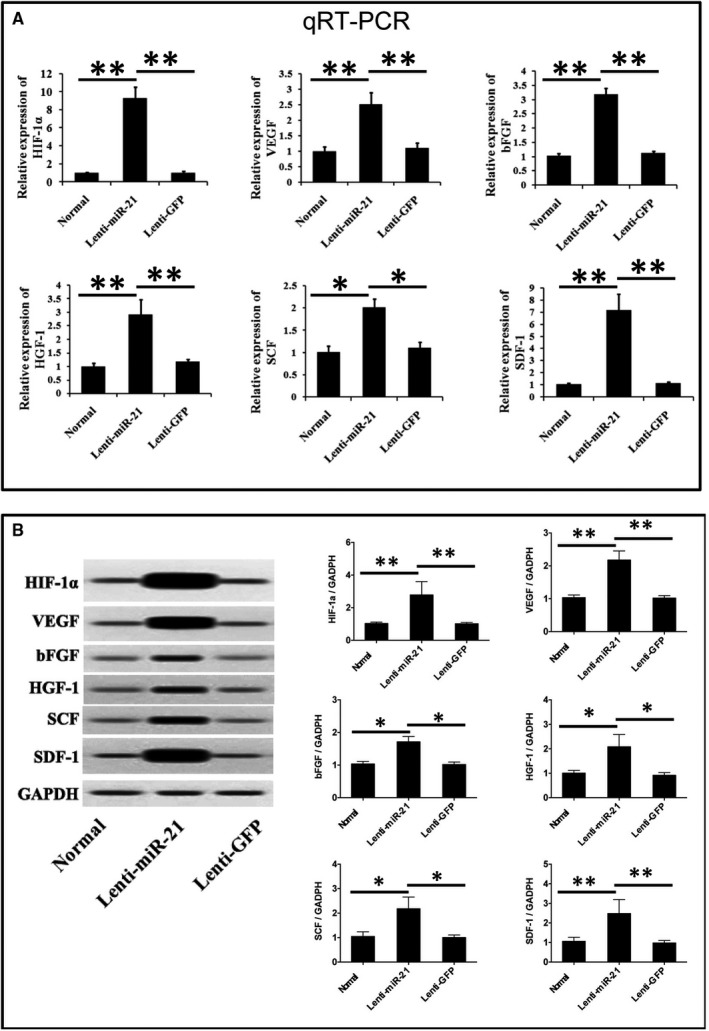
A, qRT‐PCR and (B) Western blotting evaluation of angiogenic relative proteins extents and mRNA expression. *P < 0.05, **P < 0.01

In order to study the regulation effects of miR‐21 on the angiogenic capacity of ADSCs, the angiogenic relative proteins expressions were evaluated using qRT‐PCR and Western blotting. As shown in Figure 2, as compared to the ADSCs in normal group and Lenti‐GFP group, both qRT‐PCR and Western blotting results showed that the expression levels of angiogenic factors HIF‐1α, VEGF, bFGF, HGF‐1, SCF and SDF‐1a were significantly increased in miR‐21 transfected ADSCs in Lenti‐miR‐21 group (*P* < 0.05 or *P* < 0.01).

### 3.2 Anti‐oxidative effects of miR‐21 from ROS damage

**Figure 3:**
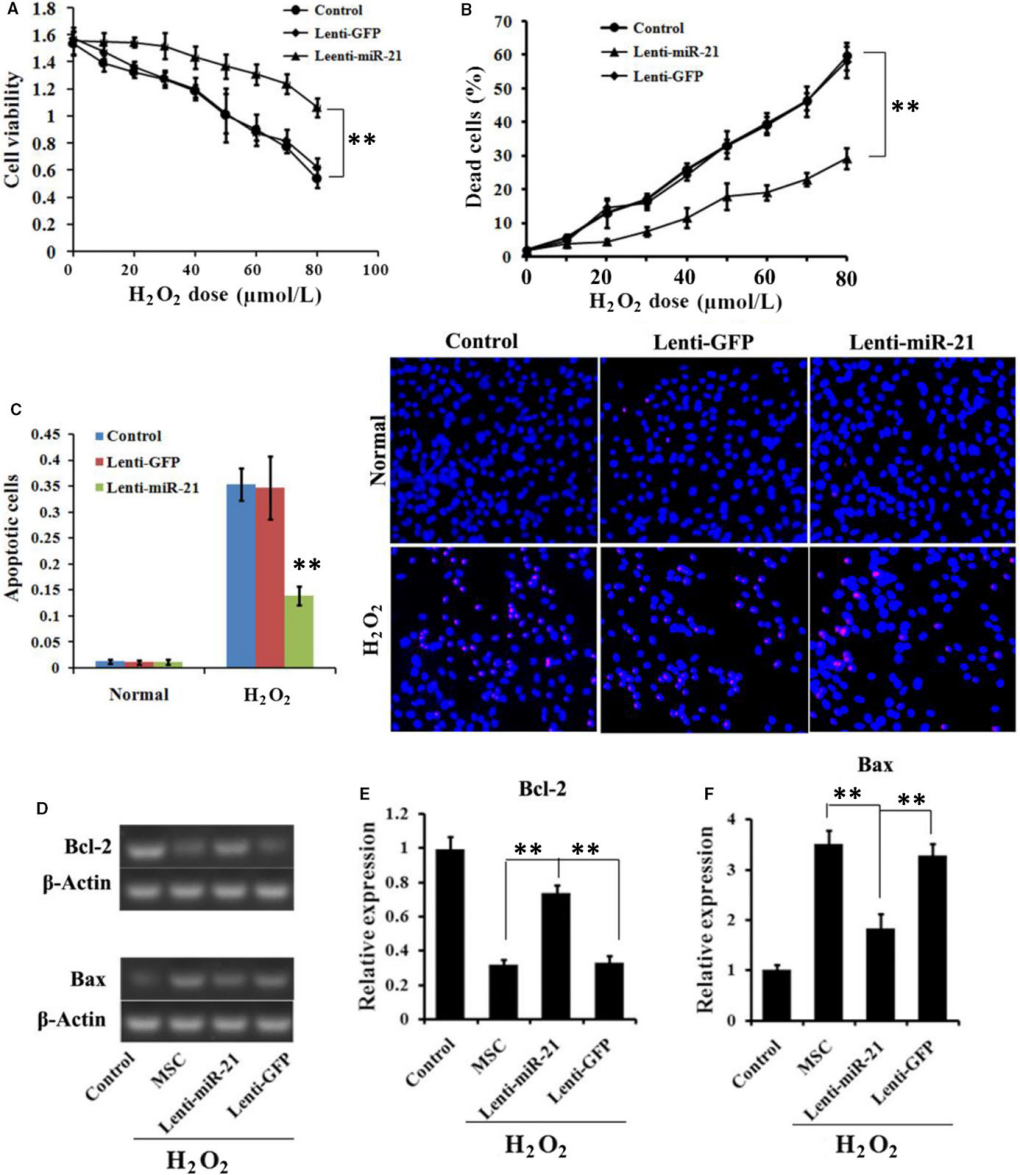
Anti‐oxidative protection role of miR‐21 modification on ADSCs. A, Cell viability of ADSCs under different concentration of H_2_O_2_ treatment. B, The proportion of dead cells under different concentration of H_2_O_2_ treatment. C, The proportion of apoptotic cells under 60 μmol/L H_2_O_2_ treatment. D, RT‐PCR and E, quantified analysis of the Bax and Bcl‐2 expression levels under normal or ROS condition. *P < 0.05, **P < 0.01

In order to further investigate the protective role of miR‐21 in oxidative stress induced injury, hydrogen peroxide (H_2_O_2_) was chosen as an exogenous ROS source to injury the ADSCs. As shown in Figure 3A, CCK‐8 results proved that between ADSCs cellular viability and H_2_O_2_ exposure concentration. The cellular viability of ADSCs from both Control group and Lenti‐GFP group decreased as the H_2_O_2_ exposure concentration increased, but the ROS damage was significantly attenuated by miR‐21 in Lenti‐miR‐21 group. Similarly, the proportion of dead ADSCs in both Control group and Lenti‐GFP group increased along with increased H_2_O_2_ concentration, which was restored via transfection of miR‐21 in Lenti‐miR‐21 group.

TUNEL calculation and staining results (Figure 3C) showed that only small proportion of ADSCs was positively for TUNEL staining in all three groups. After H_2_O_2_ treatment, the ADSCs in both Control and GFP groups were observed with increased proportion of TUNEL‐positive cells. However, the proportion was significantly decreased in Leti‐miR‐21.

RT‐PCR results (Figure 3D) showed that the H_2_O_2_ treatment could decrease relative expression levels of anti‐apoptotic executioner Bcl‐2 in Control group, but the Bcl‐2 expression level was significantly increased via miR‐21 transfection in Leti‐miR‐21 group (*P* < 0.05 or *P* < 0.01), yet not in Leti‐GFP group. H_2_O_2_ treatment could increase the mRNA expression level of critical apoptosis executioner Bax in Control group. However, after transfected with miR‐21, the Bax level was significantly decreased as compared to Control and Leti‐GFP group (*P* < 0.05 or *P* < 0.01). The results suggested that miR‐21 could relieve the oxidative stress induced injury.

### 3.3 Functional evaluation results

**Figure 4:**
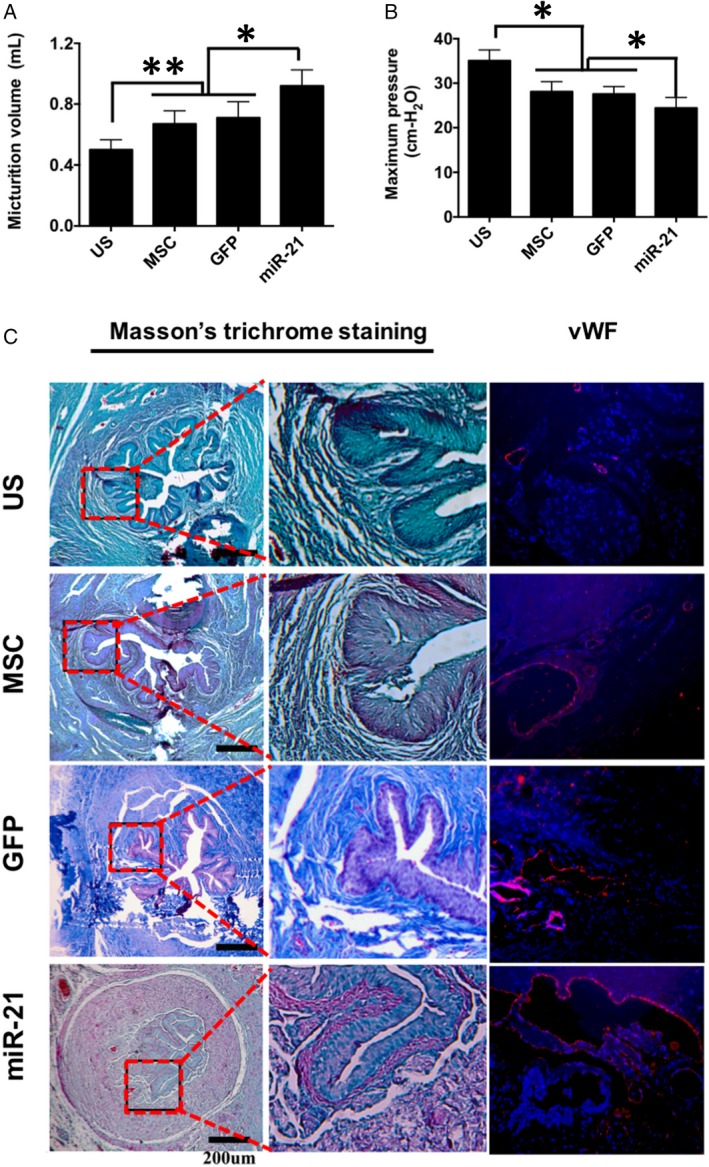
In vivo functional and histological evaluation. Measurement of the urodynamic parameters (A) micturition volume (mL) and (B) maximum pressure 4 wk after surgery. C, Masson trichrome and vWF immunofluorescence staining results. *P < 0.05, **P < 0.01

Four weeks later, the micturition volume and maximum pressure were performed to measure the urodynamic parameters of the rats with urethral stricture. As shown in Figure 4A,B, the results showed that micturition volume of MSC and GFP groups were higher than that of US group, and the mi‐R21 group was observed with highest volume. Maximum pressure results showed that MSC, GFP and miR‐21 groups significantly decreased the pressure as compared to US group (*P* < 0.05 or *P* < 0.01).

**Figure 5:**
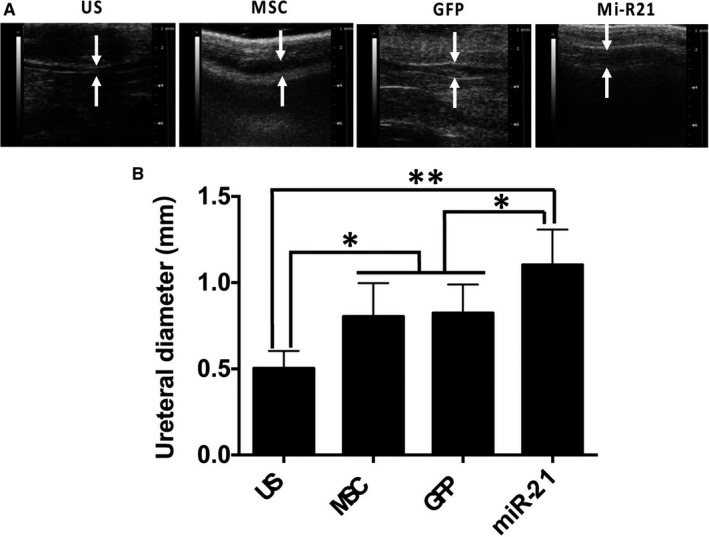
Micro‐ultrasound observation and detection of urethral diameter. A, Representative micro‐ultrasound images of rat penile urethras from different groups, and measurement of urethral diameter accordingly 4 wk after injections (n = 6), *P < 0.05, **P < 0.01

The histological analyses were also performed to evaluate the safety of ADSCs transplantation and wound healing of the urethral tissue (Figure 4C). According to macroscopic observation and histological analyses, no visible tumour was observed in ADSCs injected sites in stem cell therapy groups (Figure 4C), including MSC, GFP and miR‐21 groups, respectively. Masson's staining results of US group were observed with stroma rich in green stained collagen fibres in the spongious urethra, suggesting a high level of urethral fibrosis. The Masson and vWF staining results in US group also suggested that the epithelium and the muscle layer not fully formed yet. The levels of collagen fibres in the spongious urethra decreased after being treated with MSCs or marker gene transfected MSCs in the MSC group or GFP group. Two groups were also observed with good formation of the epithelium and the muscle layer as proved by Masson and vWF results. In the miR‐21 group, the overall structure of the spongious urethra with well formed epithelium and the muscle layer similar to that of a normal urethra was observed. The urethral structure and diameter were further evaluated by micro‐ultrasound. As shown in Figure 5, both MSC (0.83 ± 0.11 mm) and GFP (0.82 ± 0.09 mm) groups were observed with larger ureteral structure and significantly increased ureteral diameter as compared to US group (0.51 ± 0.6 mm), and the ureteral diameter was further increased in Mi‐R21 group (1.1 ± 0.12 mm) (*P* < 0.05 or *P* < 0.01). These results indicated that the transplantation of MSCs could counteract the urethral stricture formation of rats, and the therapeutic efficacy was further enhanced via miR‐21 modification.

**Figure 6:**
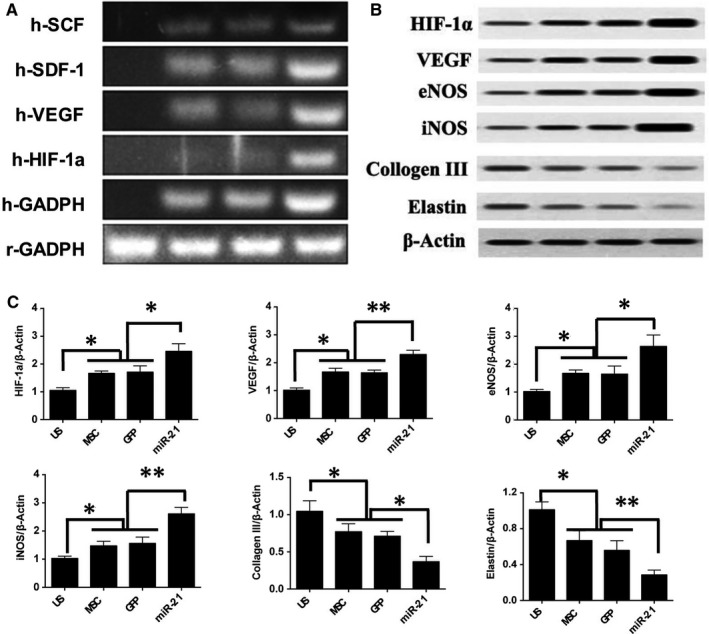
In vivo RT‐PCR and Western blotting analysis results. A, In vivo RT‐PCR measurement of the angiogenic relative genes expressions; (B) representative chemiluminescence images of blotted membranes and (C) summarized protein expression levels for HIF‐1a, VEGF, iNOS, eNOS, collagen III and elastin accordingly. *P < 0.05, **P < 0.01

RT‐PCR results (Figure 6A) confirmed high levels of angiogenic genes mRNA expression levels in miR‐21 transfected human ADSCs in Lenti‐miR‐21 group, such as HIF‐1α, VEGF, SCF and SDF‐1a. Western blotting (Figure 6B,C) results indicated that the MSCs treatment in MSC and GFP group could significantly increase contents of angiogenic proteins as compared to the US group, such as the HIF‐1a, VEGF, iNOS and eNOS. These proteins were also increased in miR‐21 group. Besides, as compared to US group, the protein contents of collagen III and elastin were decreased in MSCs transplantation groups, especially in miR‐21 group.

## 4 DISCUSSION

Studies have shown that MSC transplantation could enhance the healing and reconstitution of the urethra tissue to counteract US formation. Genetic modification of ADSCs with therapeutic genes was also proved to increase the therapeutic potential of the stem cells.^7^ The microRNA‐21 plays critical roles in the regulation of a variety of skin fibrosis, which hold great potential for new diagnostic and therapeutic options for wound remodelling and maturation.^12^, ^15^, ^22^ The present study demonstrates that miR‐21 modification in ADSCs via lentiviral transfection improved the therapeutic potential of MSCs for urethral wound healing. Furthermore, we also investigated the potential underlying mechanism involved during the healing process, with results suggesting high levels of angiogenic factors expression for accelerated vascular maturation and favourable collagen remodelling.

Urethral wound healing after injury is a complex biological process.^5^ The application of MSCs has proved to be able to improve wound healing via cell differentiation and paracrine effects. However, unfavourable microenvironment factors caused multiple impairments in cellular responses would hinder wound healing process. Among these factors contributing to impaired wound healing or excessive scar formation, the impaired production of cytokines, inflammation, oxidative stress and reduced angiogenesis are crucial. These unfavourable factors induced poor viability of MSC at the transplanted site often decreases their therapeutic potential. Thus, the improvement of the transplanted MSC survival rate was important for enhancing the secretion of factors and their biological functions in vivo. It was reported that elimination of ROS could increase the survival of the transplanted cells in the injury site with unfavourable microenvironment and eventually enhanced the stem cell‐mediated wound healing. In this study, we found that miR‐21 modification could improve survival of ADSCs under oxidative stress condition via enhancing the endogenous anti‐apoptotic activities.

Sufficient vascularization of injury tissue is imperative for adequate wound healing and remodelling. In addition to exchange of the nutrients, oxygen and growth factors, etc., blood vessels were also proved to regulate tissue/organ growth, and repair by communicating with targeted tissues via its own angiocrine signals. Increasing studies also indicated that MSCs derived angiogenic proteins could regulate the neoangiogenesis injury tissue for promoted wound healing process. Recently study also proved that miR‐21 overexpressed MSCs can effectively induce angiogenesis potential via promoting HIF‐1a activity.^24^ In the present study, in order to understand the mechanism of miR‐21 modified ADSCs in counteract urethral stricture formation, we examined the expression levels of some angiogenic genes, such as VEGF, HIF‐1a and SDF‐1a. The in vitro results demonstrated that miR‐21 overexpression in MSCs can enhance the expression of multiple target angiogenic genes. This enhanced angiogenic promotion ability may contribute to enhanced tissue regeneration and repair. As a result, the in vivo experiments suggested that the miR‐21 overexpression could increase the angiogenic genes mRNA expression levels of transplanted human ADSCs as well, and the contents of angiogenic proteins. As a result, it promoted the epithelium layer formation and thereby modified complex urethral wound healing process. However, this progression is not observed in the US group.

At the end of experiment, histological staining demonstrated that transplantation of MSCs alone contributes to good formation of the epithelium and the muscle layer as proved by Masson and vWF results, indicating that transplanted ADSCs can counteract urethral fibrosis and stricture formation, which was further enhanced via miR‐21 modification. The functional results suggested that transplantation of ADSCs, especially for miR‐21 modified ADSCs, could effectively prevented functional bladder complications caused by urethral stricture formation.

Together, these results suggested that ADSCs transplantation could effectively improve the healing and reconstitution of the urethra tissue to counteract US formation, and the miR‐21 modification via lentiviral transfection can increase the therapeutic efficacy.

### 4.1 Limitations of this study

Despite these encouraging results, there are still some limitations in the present study. Although our primary data proved that miR‐21 modification of ADSCs could increase the ADSCs' therapeutic potential for counteracting urethral stricture formation, the long‐term therapeutic effects of it need to be further verified. What's more, the main purpose of this study was to test the feasibility of miR‐21 modification for enhanced MSCs therapeutic potential for the healing and reconstitution of the urethra postoperatively, which could potentially be used to limit urethral stricture recurrence after urethrotomy. However, if local injection of ADSC, especially for gene modified ADSCs, could be used for treatment of urethral stricture that had been formed still need to be tested. What's more, although our results indicated the regulation effects of miR‐21 modified ADSC on stem cell survival, ROS scavenging and angiogenesis, it is still insufficient to completely understand the whole roles of ADSCs counteracting urethral stricture formation, as well as the underlying cellular and molecular mechanism. Because urethral wound healing after injury is a complex biological process and involved many other cell types, such as inflammatory response and relative cells. The immunomodulatory benefits of miR‐21 modification on ADSC as well as the molecular mechanism or signalling pathways involved in it is under investigation.

## 5 CONCLUSION

The present study demonstrates that miR‐21 modification in ADSCs could improve urethral wound healing microenvironment, enhance stem cell survival through ROS scavenging and promote the neovascularization via regulating angiogenic genes expression, which eventually increase the ADSCs' therapeutic potential for urethral wound healing. The current findings may offer new wide‐reaching implications for the future of substitution urethroplasty and overcome recurrence of strictures after urethrotomy.

## CONFLICT OF INTEREST

The authors confirm that there is no conflict of interests.
