# Supplementary material for: miR‐21 modification enhances the performance of adipose tissue‐derived mesenchymal stem cells for counteracting urethral stricture formation
Source: J Cell Mol Med. 2018 Sep 4;22(11):5607–16. doi: 10.1111/jcmm.13834 (PMC6201219; doi:10.1111/jcmm.13834)
Supplement: Supplementary file 1 [file JCMM-22-5607-s001.docx]

**Supporting Information**

**miR-21 modification enhances the performance of adipose tissue-derived mesenchymal stem cells for counteracting urethral stricture formation**

Zongcheng Feng*, Hongrun Chen, Taozhu Fu, Lianfeng Zhang, Yushan Liu

Department of Urology, No. 731 Hospital of China Aerospace Science & Industry Corporation；

* Corresponding author: Zongcheng Feng, Department of Urology, No. 731 Hospital of China Aerospace Science & Industry Corporation, Beijing 100074, China; Tel. +86-01-68374065 E-mail address: fzch1965@126.com;

**Table S1**. Primers used for qRT-PCT in the present study.

| Genes | Primers |
| --- | --- |
| h-VEGF | 5’- CCTTGCTGCTCTACCTCCAC -3’ |
|  | 5’- GCAGTAGCTGCGCTGATAGA-3’ |
| h-HGF-1 | 5’- AAACGCAAACAGGTTCTCAATG -3’ |
|  | 5’- CTATGACTGTGGTACCTTATATG -3’ |
| h-HIF-1a | 5’- CATCTCCATCTCCTACCCACAT -3’ |
|  | 5’- ACTCCTTTTCCTGCTCTGTTTG -3’ |
| h-bFGF | 5’- CTGGCTATGAAGGAAGATGGA -3’ |
|  | 5’- TGCCCAGTTCGTTTCAGTG- -3’ |
| h-SCF | 5’- ATGAAGAAGACACAACTTG-3’ |
|  | 5’- AAG- GCATCAATGGATCTATT- -3’ |
| h-SDF-1 | 5’- GCCATGAACGCCAAGGTCGTGGT -3’ |
|  | 5’-CCTCGAGTGGGTCTAGCGGAAAG -3’ |
| h-Bcl-2 | 5’-ACACTTTATGTGTGGTTAGAAAGGG-3’ |
|  | 5’- ACCTGGATCTTTTCTAACAGGATG -3’ |
| h-Bax | 5’- GGCCCACCAGCTCTGAGCAGA-3’ |
|  | 5’- GCCACGTGGGCGGTCCCAAAGT -3’ |
| hGAPDH | 5’- CTCTGACTTCAACAGCGACA -3’ |
|  | 5’- TCTCTCTCTTCCTCTTGTGC -3’ |
| rGADPH | 5’- CATGGCCTTCCGTGTTCCTA -3’ |
|  | 5’- CCTGCTTCACCACCTTCTTGAT- -3’ |
